# Sequential Cross-Sectional Surveys in Orange Farm, a Township of South Africa, Revealed a Constant Low Voluntary Medical Male Circumcision Uptake among Adults despite Demand Creation Campaigns and High Acceptability

**DOI:** 10.1371/journal.pone.0158675

**Published:** 2016-07-18

**Authors:** Esaie Marshall, Reathe Rain-Taljaard, Motlalepule Tsepe, Cornelius Monkwe, Florence Hlatswayo, Simphiwe Tshabalala, Simphiwe Khela, Lindo Xulu, Dumazile Xaba, Tebogo Molomo, Thobile Malinga, Adrian Puren, Bertran Auvert

**Affiliations:** 1 Institut National de la Santé et de la Recherche Médicale, Unit 1018, Centre for Research in Epidemiology and Population Health, Villejuif, France; 2 Faculty of medicine, Research Unit 4184, Faculty of Medicine, University of Burgundy, Dijon, France; 3 Progressus Research and Development, Johannesburg, South Africa; 4 National Institute for Communicable Diseases, National Health Laboratory Service, Johannesburg, South Africa; 5 Faculty of Health Sciences, University of the Witwatersrand, Johannesburg, South Africa; 6 Assistance Publique—Hôpitaux de Paris, Hôpital Ambroise Paré, Boulogne-Billancourt, France; 7 Faculty of Medicine, University of Versailles-Saint Quentin, Versailles, France; University of Pittsburgh, UNITED STATES

## Abstract

**Background:**

WHO recommends a male circumcision (MC) prevalence rate higher than 80% to have a substantial impact on the HIV-AIDS epidemic in Eastern and Southern Africa. Orange Farm, a township in South Africa, has a free-for-service voluntary medical male circumcision (VMMC) clinic in operation since 2008. Following an intense campaign from 2008 to 2010, MC prevalence rate increased to 55.4% (ANRS-12126). Ongoing and past VMMC campaigns focused on youths, through school talks, and adults at a community level. The main objective of the study was to assess the change in MC prevalence rate among adults aged 18–19 and 18–49 years in the past 5 years.

**Methods:**

A cross-sectional survey (ANRS-12285) was conducted among a random sample of 522 adult men in 2015. MC status and characteristics of participants were collected through a genital examination and a face-to-face questionnaire.

**Results:**

MC prevalence rate among young adult men aged 18–19 years increased markedly from 61.2% (95%CI: 57.4% to 65.0%) in 2010 to 87.5% (76.0% to 94.6%) in 2015 (p<0.001). In the same period, among men aged 18–49 years, MC prevalence rate varied slightly from 55.4% (53.6% to 57.1%) to 56.7% (52.4% to 60.9%). In 2015, 84.9% (79.2% to 89.5%) of uncircumcised adult men reported that they were willing to be circumcised. However, we estimated that only 4.6% (11/237; 2.5% to 7.9%) of the uncircumcised men underwent circumcision in 2015, despite 117/185 (63.2%; 95%CI: 56.1% to 69.9%) who reported that they were definitely willing to become circumcised.

**Conclusions:**

In Orange Farm, VMMC campaigns were successful among the youth and led to a sufficiently high MC prevalence rate to have a substantial impact in the future on the HIV-AIDS epidemic. However, despite high acceptability and a free VMMC service, VMMC campaigns since 2010 have failed to increase MC prevalence rate among adults to above 80%. These campaigns should be revisited.

## Introduction

Three randomized controlled trials have shown that male circumcision (MC) reduces the risk of heterosexual transmission of HIV infection to men by approximately 60% [1–3]. Following these trials, WHO and UNAIDS recommended in 2007 the use of voluntary medical male circumcision (VMMC) to fight the HIV-AIDS epidemic in countries with a low MC prevalence rate and a high HIV prevalence rate [4], conditions which correspond to most of the countries of Eastern and Southern Africa. A MC prevalence rate of 80% was recommended by WHO and UNAIDS as the target required to have a substantial impact on the HIV-AIDS epidemic [5].

Following the WHO and UNAIDS recommendation, a list of 14 priority countries in Africa was established and the rollout of VMMC was implemented, funded in the most part by PEPFAR. Over time, the MC prevalence in these countries increased, but coverage, which is the MC prevalence rate, varies substantially between countries. As of December 2014, while Ethiopia, Kenya and Tanzania have already reached 80% coverage targets, Lesotho, Malawi, Namibia, Rwanda and Zimbabwe have low coverage, ranging from 6% to 26% [6]. It is uncertain whether an 80% MC prevalence rate will be reached in all the priorities countries.

The township of Orange Farm, located in South Africa, one of the priority countries, has been a pioneer in VMMC. It was the study area where the first randomized controlled circumcision trial (ANRS-1265) was conducted and, to the best of our knowledge, where the first VMMC rollout (ANRS-12126) was implemented in the period 2008–2010. This rollout led to an increase in the adult MC prevalence rate from 12% in 2008 to 53% in 2011 [7]. Since 2010, and similar to many other places in Africa, routine VMMC demand creation has been organized, through school talks, advertisements and periodic door-to-door distribution of fliers. The main objective of this study was to assess the changes in MC prevalence rate in the past 5 years among men aged 18–49 years and to assess whether an 80% coverage rate had been reached. The secondary objectives were to identify demographic characteristics of uncircumcised men, determine knowledge and attitudes associated with MC, and to estimate the annual uptake of MC in the community.

## Methods

### Study setting

This study was conducted in the township of Orange Farm, in which the adult population is estimated to be 110,000 residents. The township is located approximately 40km from Johannesburg, in the Gauteng province. The province has experienced one of the most severe HIV epidemics in the world, with HIV prevalence estimated at 30% among antenatal women in 2011 [8]. In this township, a clinic offering free-for-service VMMC has been operating since 2008. It accepts males from the age of 10 years old.

### Surveys

A cross-sectional biomedical survey (ANRS-12285) was conducted among a random sample of men between August and September 2015. A first random sample of 34 clusters was obtained among the 346 clusters obtained from Statistics South Africa Enumerator Area aerial photographs. In each of the 34 clusters, a systematic sample of 1 in 5 of the households was selected [9]. Voluntary, written informed consent was obtained from men aged 18–49 years. All men who stayed at least 2 nights in the household in the past 7 days and who spoke English or one of the 2 main local languages (Sesotho and IsiZulu) were eligible. Each participant was interviewed at home using an anonymous, structured and standardized questionnaire. Interviews were followed by individual and confidential HIV and STI counselling and testing. Participants underwent a clinical examination performed by a trained male nurse to assess their clinical MC status (presence or absence of foreskin). Participants with symptomatic STIs were treated free of charge at the study site or at local health facilities according to the national STI syndromic management treatment guidelines. Individuals who tested positive for HIV were referred for appropriate care and treatment. Among 981 eligible households, 870 accepted to participate in the study, which corresponds to a response rate of 88.5%. In these 870 households, 696 men were eligible and 522 men accepted to participate in the study, which corresponds to a response rate of 75.0%.

During the survey, the following background characteristics were collected; age group, ethnic group, religion, alcohol consumption, education level, occupation, current and previous marital status, parenthood status, monthly income and length of stay in Orange Farm. Other collected factors were HIV testing history and self-assessment of HIV infection risk level. Factors related to knowledge and attitude regarding MC were also collected. For men reporting themselves as uncircumcised, willingness to become circumcised and the main reason for not being circumcised were collected. For circumcised men, we collected the main reason for being circumcised and the date of circumcision. We observed that some men made false claims of being circumcised. As a result and because the collection of data preceded the genital examination, these men were excluded from the data collected specifically from uncircumcised men (willingness to become circumcised and main reason for being uncircumcised).

The Human Research Ethics Committee (Medical) of the University of the Witwatersand, Johannesburg, South Africa, granted ethical clearance for the study on January 19^th^ 2015 (M140946).

### Statistical analysis

The main outcome of this study was the MC status observed by the nurse during the genital examination. Analyses were conducted among the whole sample, and among the circumcised and uncircumcised men. We analyzed the association of background characteristics with MC status by estimating prevalence rate ratios (PRR) and adjusted PRR (aPRR) using univariate and multivariate general linear models (Poisson regression). We used the binomial test to compare observed male circumcision prevalence rates with the value of 80%. 95%CI of proportions were calculated using the Clopper-Pearson interval by calculating quantiles from the beta distribution [10]. A sample size of 1000 households was judged as sufficient to obtain a sample of at least 500 men. Analyses were computed using the R statistical package [11].

## Results

### Characteristics of survey participants

Among the 522 men taking part in the survey, the mean age (median; interquartile range) was 30.5 years (28.9; 23.0 to 37.1). The prevalence rate of MC was 296/522 (56.7%; 95%CI: 52.4% to 60.9%), which is significantly different from 80% (p<0.001).

Table 1 indicates the background characteristics of the sample. Approximately 75% of the participants reported being residents of Orange Farm for five years or longer. The Sesotho and IsiZulu ethnic groups each represent one third of the population. More than half of the participants reported to be employed and half of the participants left school without having completed the 12^th^ grade.

**Table 1:** Background characteristics of the sample surveyed in 2015 and association with male circumcision status.

Background characteristics	Uncircumcised	Circumcised	Total	PRR	aPRR
	N = 226	N = 296	N = 522	(95%CI) p-value	(95%CI) p-value
	n (%)	n (%)	n (%)		
**Age group (%)**					
18–24	45 (19.9%)	131 (44.3%)	176 (33.7%)	1 p = 0.000	1 p = 0.000
25–34	93 (41.2%)	98 (33.1%)	191 (36.6%)	1.90 (1.46–2.49) p = 0.000	1.77 (1.28–2.43) p = 0.001
35–49	88 (38.9%)	67 (22.6%)	155 (29.7%)	2.22 (1.69–2.91) p = 0.000	2.03 (1.42–2.91) p = 0.000
**Ethnic group (%)**					
Sotho	73 (32.3%)	98 (33.1%)	171 (32.8%)	1 p = 0.029	1 p = 0.016
Zulu	90 (39.8%)	88 (29.7%)	178 (34.1%)	1.18 (0.94–1.50) p = 0.160	1.21 (0.95–1.55) p = 0.120
Other	63 (27.9%)	110 (37.2%)	173 (33.1%)	0.85 (0.66–1.10) p = 0.220	0.84 (0.65–1.09) p = 0.180
**Religion (%)**					
Christian	67 (29.6%)	91 (30.7%)	158 (30.3%)	1 p = 0.675	Not included
No religion	87 (38.5%)	103 (34.8%)	190 (36.4%)	1.08 (0.85–1.37) p = 0.530	
Other	72 (41.4%)	102 (34.5%)	174 (33.3%)	0.98 (0.76–1.25) p = 0.850	
**Alcohol consumption (%)**					
Less than once a week	154 (68.1%)	203 (68.6%)	357 (68.4%)	1	Not included
Once a week or more	72 (31.9%)	93 (31.4%)	165 (31.6%)	1.01 (0.82–1.25) p = 0.920	
**Education level (%)**					
Not at school and grade 12 not completed	141 (62.4%)	128 (43.2%)	269 (51.5%)	1 p = 0.000	1 p = 0.072
At school and grade 12 not completed	16 (7.1%)	52 (17.6%)	68 (13.0%)	0.45 (0.30–0.66) p = 0.000	0.74 (0.46–1.19) p = 0.220
Grade 12 completed	69 (30.5%)	116 (39.2%)	185 (35.4%)	0.71 (0.57–0.88) p = 0.002	0.78 (0.62–0.98) p = 0.032
**Occupation (%)**					
Employed	148 (65.5%)	161 (54.4%)	309 (59.2%)	1 p = 0.004	1 p = 0.696
Unemployed	52 (23.0%)	68 (23.0%)	120 (23.0%)	0.90 (0.71–1.15) p = 0.410	0.98 (0.73–1.33) p = 0.920
Other	26 (11.5%)	67 (22.6%)	93 (17.8%)	0.58 (0.43–0.80) p = 0.001	0.85 (0.59–1.24) p = 0.400
**Having ever been married (%)**					
No	89 (39.4%)	159 (53.7%)	248 (47.5%)	1	1
Yes	137 (60.6%)	137 (46.3%)	274 (52.5%)	1.39 (1.14–1.70) p = 0.001	1.06 (0.81–1.38) p = 0.690
**Having at least one child (%)**					
No	80 (35.4%)	140 (47.3%)	220 (42.1%)	1	1
Yes	146 (64.6%)	156 (52.7%)	302 (57.9%)	1.33 (1.08–1.63) p = 0.007	0.84 (0.64–1.10) p = 0.210
**Monthly incomes in ZAR**^a^					
Less than 1000	102 (45.1%)	154 (52.0%)	256 (49.0%)	1	1
More than or equal to 1000	124 (54.9%)	142 (48.0%)	266 (51.0%)	1.17 (0.96–1.43) p = 0.120	0.99 (0.76–1.28) p = 0.930
**In Orange Farm more than 5 years**					
No	69 (30.5%)	60 (20.3%)	129 (24.7%)	1	1
Yes	157 (69.5%)	236 (79.7%)	393 (75.3%)	0.75 (0.60–0.92) p = 0.008	0.76 (0.61–0.94) p = 0.013

^a^ cut-off value was the median value

CI: confidence interval

PRR: prevalence rate ratio obtained using Poisson regression

aPRR: adjusted PRR on the covariates in the table having a univariate p-value ≤ 0.20

ZAR: South African Rand

Table 2 indicates the other characteristics of the participants. Approximately 75% of uncircumcised men knew about free-of-charge circumcision in Orange Farm. The majority of the participants had good knowledge regarding MC and preferred to have their male children circumcised. For example, nearly all men reported that a circumcised man can become infected with HIV and that a woman having unprotected sex with a circumcised man is not protected against HIV infection.

**Table 2:** Characteristics related to HIV and male circumcision of the sample surveyed in 2015 and association with male circumcision status.

Characteristics	Uncircumcised	Circumcised	Total	PRR	aPRR
	N = 226	N = 296	N = 522	(95%CI) p-value	(95%CI) p-value
	n (%)	n (%)	n (%)		
**Has been tested for HIV**					
No	70 (31.0%)	42 (14.2%)	112 (21.5%)	1	1
Yes	156 (69.0%)	254 (85.8%)	410 (78.5%)	0.61 (0.49–0.75) p = 0.000	0.58 (0.47–0.72) p = 0.000
**Reported himself as at risk of HIV infection**					
No	141 (62.4%)	211 (71.3%)	352 (67.4%)	1	1
Yes	85 (37.6%)	85 (28.7%)	170 (32.6%)	1.25 (1.02 to 1.52) p = 0.029	1.17 (0.95–1.45) p = 0.130
**Knows that MC can be done for free in OF**					
No	54 (23.9%)	20 (6.8%)	74 (14.2%)	1	1
Yes	172 (76.1%)	276 (93.2%)	448 (85.8%)	0.53 (0.42–0.66) p = 0.000	0.64 (0.50–0.83) p = 0.001
**Male circumcision must be done in winter**					
No	85 (37.6%)	101 (34.1%)	186 (35.6%)	1	1
Yes	141 (62.4%)	195 (65.9%)	336 (64.4%)	0.92 (0.75–1.13) p = 0.410	0.91 (0.74–1.12) p = 0.380
**Most women prefer circumcised men**					
Disagree or don't know	80 (35.4%)	51 (17.2%)	131 (25.1%)	1	1
Agree	146 (64.6%)	245 (82.8%)	391 (74.9%)	0.61 (0.50–0.75) p = 0.000	0.65 (0.53–0.81) p = 0.000
**Circumcision increases pleasure during sex**					
Disagree or don't know	96 (42.5%)	50 (16.9%)	146 (28.0%)	1	1
Agree	130 (57.5%)	246 (83.1%)	376 (72.0%)	0.53 (0.43–0.64) p = 0.000	0.57 (0.46–0.69) p = 0.000
**Circumcised men do not need to use condoms to protect themselves from HIV**					
Agree or don't know	24 (10.6%)	7 (2.4%)	31 (5.9%)	1	1
Disagree	202 (89.4%)	289 (97.6%)	491 (94.1%)	0.53 (0.39–0.73) p = 0.000	0.64 (0.46–0.89) p = 0.008
**Circumcised men can become infected with HIV**					
Disagree or don't know	20 (8.8%)	12 (4.1%)	32 (6.1%)	1	1
Agree	206 (91.2%)	284 (95.9%)	490 (93.9%)	0.67 (0.48–0.95) p = 0.025	0.70 (0.49–0.99) p = 0.047
**Male circumcision protects woman from HIV**					
No	219 (96.6%)	296 (100.0%)	515 (98.7%)	1	1
Yes	7 (3.1%)	0 (0.0%)	7 (1.3%)	2.35 (1.33–4.15) p = 0.003	2.13 (1.19–3.83) p = 0.012
**Circumcised men are protected from HIV**					
No	215 (95.1%)	286 (96.6%)	501 (96.0%)	1	1
Yes	11 (4.9%)	10 (3.4%)	21 (4.0%)	1.22 (0.77–1.93) p = 0.390	1.42 (0.89–2.25) p = 0.140
**Prefer child to be circumcised**					
No	15 (6.6%)	1 (.3%)	16 (3.1%)	1	1
Yes	211 (93.4%)	295 (99.7%)	506 (96.9%)	0.44 (0.30–0.66) p = 0.000	0.45 (0.30–0.68) p = 0.000

CI: confidence interval

PRR: prevalence rate ratio obtained using Poisson regression

aPRR: adjusted PRR on the covariates of the Table 1 having a univariate p-value ≤ 0.20

### MC prevalence rate over time

**Fig 1:**
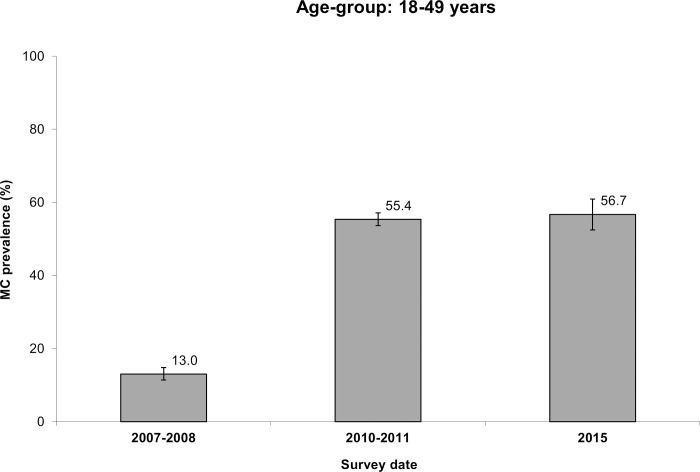
Male circumcision (MC) prevalence rate among men aged 18–49 years obtained in three independent cross-sectional surveys conducted in Orange Farm in the years 2007–2008, 2010–2011 and 2015.

Fig 1 represents the variation with time of the MC prevalence rate among adults aged 18 to 49 years. This prevalence rate increased only very slightly between 2010–2011 and 2015, from 55.4 to 56.7% (p = 0.60). However, when controlling for age group, we found an aPRR of 1.15 (95%CI: 1.05 to 1.25) p = 0.002. Knowing that the time since the 2010–2011 survey was 4.5 years, this aPRR corresponds to an average increase per year of 3.1% (95%CI: 1.2% to 8.6%).

**Fig 2:**
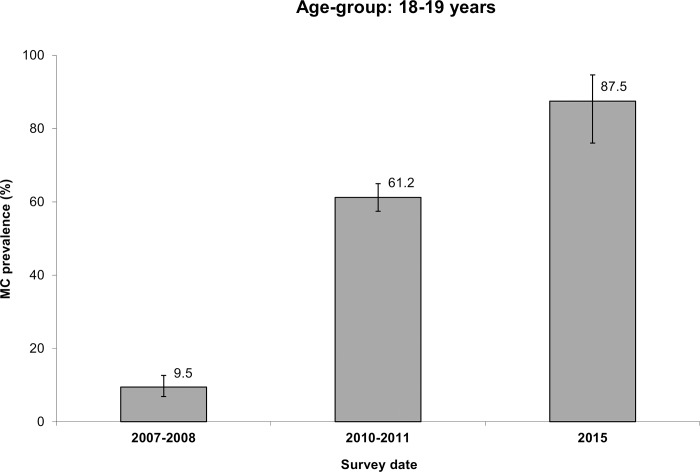
Male circumcision (MC) prevalence rate among men aged 18–19 years obtained in three independent cross-sectional surveys conducted in Orange Farm in the years 2007–2008, 2010–2011 and 2015.

Fig 2 represents the variation with time of the MC prevalence rate among adults aged 18 to 19 years. This prevalence rate increased from 9.5% in 2007–2008 to 87.5% (42/48; 95%CI: 76.0% to 94.6%) in 2015, a value not significantly different from 80% (p = 0.28).

Among adults aged 20 to 29 years the MC prevalence increased from 56.9% (1146/2013) to 61.8% (144/233) between 2010–2011 and 2015, which corresponds to an aPRR adjusted on age group of 1.10 (0.98–1.23) p = 0.10.

Among the circumcised men, 11 reported to have been circumcised in the past 12 months. The proportion of circumcised men among those who were uncircumcised 12 months ago was estimated as 11/(226+11), corresponding to an uptake rate of 4.6% (95%CI: 2.5% to 7.9%) per year.

### Reported MC status

Among the 337 men who reported to be circumcised, 41 (12.2%; 95%CI: 9.0% to 16.0%) were in fact uncircumcised. Among the 185 men who reported to be uncircumcised 0 (95%CI: 0.0% to 1.3%) were in fact circumcised. To report having attended an initiation school was not a good marker of the MC status as 88/131 (67.2%; (95%CI: 58.8% to 74.8%) were circumcised, while the number of circumcised men among those reporting to not have attended an initiation school was 208/391 or 53.2% (95%CI: 48.2% to 58.1%).

### Characteristics of uncircumcised men

As shown in Table 1, in comparison with circumcised men, uncircumcised men were older, more often Zulu in ethnic origin, less educated and more often recently resident in Orange Farm. The multivariate analysis indicated in Table 2 shows that among uncircumcised men, in comparison with circumcised men, the proportion having ever been tested for HIV was 42% lower, the proportion reporting being at risk of HIV infection was 17% higher, the proportion reporting that MC increases pleasure during sex was 43% lower, the proportion reporting that circumcised men do not need to use condoms to protect them from HIV was also 36% lower, and the proportion reporting that circumcised men can become infected with HIV was 30% lower.

Table 3 summarizes the main reasons reported by uncircumcised men for not being circumcised. 65.4% of the reasons were accounted for by the following responses: "It is not my culture", "Fear of the procedure, fear of pain or injury", and "I do not have the time".

**Table 3:** Main reason given by uncircumcised men for not being circumcised.

Main reported reason for not being circumcised	n (N = 185)	%	95% CI
It is not my culture	51	27.6%	21.5% to 34.3%
Fear of the procedure or fear of pain or injury	37	20.0%	14.7% to 26.2%
I do not have the time	33	17.8%	12.8% to 23.8%
No specific reasons or Don't know	22	11.9%	7.8% to 17.1%
It is against my religion	9	4.9%	2.4% to 8.7%
Lack of knowledge about male circumcision	8	4.3%	2.1% to 8.0%
My family is opposed	7	3.8%	1.7% to 7.3%
Do not see the need	5	2.7%	1.0% to 5.8%
Too old	3	1.6%	0.5% to 4.3%
Willing to be traditionally circumcised	2	1.1%	0.2% to 3.4%
Discomfort with female staff	1	0.5%	0.1% to 2.5%
The abstinence period is too long	1	0.5%	0.1% to 2.5%
Waiting to be circumcised	1	0.5%	0.1% to 2.5%
Oppose to medical circumcision	1	0.5%	0.1% to 2.5%
Lack of pressure from relative	1	0.5%	0.1% to 2.5%
My partner is opposed	0	0.0%	0.0% to 1.3%
The procedure/wait time is too long	0	0.0%	0.0% to 1.3%
No transportation	0	0.0%	0.0% to 1.3%
Not the right season to become circumcised	0	0.0%	0.0% to 1.3%
Too expensive	0	0.0%	0.0% to 1.3%
Other reason	3	1.6%	0.5% to 4.3%

CI: confidence interval

Among the 185 uncircumcised men, 117 (63.2%; 95%CI: 56.1% to 69.9%) reported that they were "Definitely" willing to become circumcised in the future, and 40 (21.6%; 95%CI: 16.2% to 28.0%) said "Maybe". As a result, a total of 157/185 84.9%; 95%CI: 79.2% to 89.5%) were not opposed to undergoing circumcision. Six uncircumcised men reported that they were "Unlikely" (3.2%; 95%CI: 1.4% to 6.6%) and 22 reported as being "Definitively not" (11.9%; 95%CI: 7.8% to 17.1%) willing to undergo circumcision in the future. Among those 28 men not willing to undergo circumcision, 15 reported that their culture or religion was the main reason for not being circumcised (53.6%; 95%CI: 35.5% to 70.9%).

### Main reason for being circumcised

The main reasons for being circumcised are given in Table 4. Approximately 63% of the participants reported the three following main reasons "To reduce the risk of getting HIV", "Because of tradition or religion" and "Hygiene".

**Table 4:** Main reason given by circumcised men for being circumcised.

Main reason for being circumcised	n (N = 296)	%	95% CI
To reduce the risk of getting HIV	130	43.9%	38.3% to 49.6%
Because of tradition or religion	61	20.6%	16.3% to 25.5%
Hygiene	51	17.2%	13.3% to 21.8%
Peer pressure	15	5.1%	3.0% to 8.0%
To become a man	6	2.0%	0.9% to 4.1%
Parental decision	6	2.0%	0.9% to 4.1%
To avoid pain or problems during sex	4	1.4%	0.5% to 3.2%
Medical MC to avoid traditional MC	3	1.0%	0.3% to 2.7%
No specific reason	3	1.0%	0.3% to 2.7%
My wife or girlfriend asked me to do so	2	0.7%	0.1% to 2.1%
Because it is necessary before getting married	2	0.7%	0.1% to 2.1%
For sex pleasure	2	0.7%	0.1% to 2.1%
To solve a penis problem	2	0.7%	0.1% to 2.1%
Don't know	1	0.3%	0.0% to 1.6%
Did not like his uncircumcised penis	1	0.3%	0.0% to 1.6%
Other	7	2.4%	1.1% to 4.6%

CI: confidence interval

MC: male circumcision

## Discussion

### Main results

Using sequential, independent and random cross-sectional surveys conducted over a period of 7.5 years in the township of Orange Farm, we observed that the prevalence rate of MC among adults aged 18–49 remained almost stable between 2010–2011 and 2015. However, the late adolescent MC prevalence rate among men aged 18–19 years was just over 80%. The vast majority of the uncircumcised adults reported that they would be willing to undergo circumcision.

### Trends over time

In Orange Farm, an 80% MC coverage has already been achieved among young adults. This high coverage among young adults likely results from the intense campaigns among schools that was started with the Bophelo Pele project in 2008 [12] and repeated each year. The campaigns have led to a change of social norms regarding MC among the youth. To sustain the high coverage of MC among the youth, school talks and the free VMMC clinic in Orange Farm must be maintained. The reason for this success among the youth may be the fact that MC is now culturally and socially more acceptable for the youth than for adults [13,14].

A consequence of the high MC prevalence rate among the youth is that MC prevalence rate is expected to increase among adults with the replacement of generations. This increase will, however, take time. Our study showed that the increase among adults is unlikely to be expected from the current VMMC demand creation in Orange Farm, as the MC prevalence rate has remained relatively stable since 2011. The current demand creation is less intensive than the initial implementation during the 2008–2010 period. For example, between 2008 and 2010 thirty-six field workers were recruiting men for VMMC in Orange Farm. After 2010 this was reduced to two or three.

The results obtained in Orange Farm cannot be immediately extrapolated to other settings. Orange Farm is, however, considered as having similar characteristics to other townships in South Africa. The fact that MC prevalence rate is stable among adults despite the history of VMMC, some ongoing VMMC demand creation activities and the presence of a free-for-service VMMC clinic in the township is not reassuring. Coverage of 80% among adults may be difficult to obtain in South Africa.

In order to obtain MC coverage of 80% in the 14 priority countries, 20.8 million VMMCs will have to be performed [5]. To have reached the target mid-point of 10.4 million MCs is a remarkable success [15] obtained mainly thanks to PEPFAR funding but also due to political investment in these countries. 80% MC coverage has already been obtained in Ethiopia, Kenya and Tanzania. The experience in Orange Farm draws attention to the challenges in achieving the required coverage of 80% in some settings.

### Barriers to MC program success

The important factors associated with being uncircumcised were age and ethnic group. Such factors have already been found to be important in other studies conducted in Eastern and Southern African countries where MC prevalence was low in the 2000s [16–20]. As also found in several other studies, the main reported reasons for not being circumcised were related to culture and fear of pain. Reasons that may be relatively easily addressed are fear of pain and lack of time, which represented almost 36% of the reasons mentioned by the participants [16,18,19,21].

### Limitations

We acknowledge that our study has two limitations that need to be considered. Firstly, the response rate among participants was approximately 75%. The fieldworkers reported that about half of the reasons given for refusing to participate in the survey were related to the necessity to have a genital examination. The potential bias could not be assessed because no data were collected among those who refused to participate in the survey. Such a response rate is common among cross-sectional surveys. Secondly, we observed that 12% of men made false claims of being circumcised. As a result, and described in the Methods section, the analyses of the willingness to become circumcised and the main reason for being uncircumcised were conducted among 185 men only instead of 226.

## Conclusion

Our study showed that uncircumcised men have accurate knowledge about MC, which shows that convincing uncircumcised men to become circumcised is not a question of diffusing knowledge. To obtain coverage of 80% from the current MC prevalence rate of 56%, approximately 55% (24/100-56) of uncircumcised men must be convinced to undergo circumcision. This should be feasible as our study showed that 63% of uncircumcised men reported that they were "definitely" willing to become circumcised. However, it is unlikely that the current methods of demand creation used to encourage circumcision will be successful in achieving the 80% coverage among adult males in the near future. Indeed, as shown in this study, approximately 75% of the uncircumcised men interviewed for this survey in Orange Farm had lived in this community for more than 5 years, and thus were exposed to the intense campaign of 2008–2010 and the subsequent campaigns from 2011. In this context, the current VMMC campaigns among adults in Orange Farm should be revisited and new approaches devised and tested to convince uncircumcised men to undergo circumcision. Among these new possible approaches, motivational talks and financial incentives should be considered [22–24].

## References

[1] Auvert B, Taljaard D, Lagarde E, Sobngwi-Tambekou J, Sitta R, Puren A. Randomized, controlled intervention trial of male circumcision for reduction of HIV infection risk: the ANRS 1265 Trial. PLoS Med. 2005;2: e298 16231970 10.1371/journal.pmed.0020298 PMC1262556

[2] Bailey RC, Moses S, Parker CB, Agot K, Maclean I, Krieger JN, et al Male circumcision for HIV prevention in young men in Kisumu, Kenya: a randomised controlled trial. Lancet. 2007;369: 643–56. 17321310 10.1016/S0140-6736(07)60312-2

[3] Gray RH, Kigozi G, Serwadda D, Makumbi F, Watya S, Nalugoda F, et al Male circumcision for HIV prevention in men in Rakai, Uganda: a randomised trial. Lancet. 2007;369: 657–66. 17321311 10.1016/S0140-6736(07)60313-4

[4] WHO-UNAIDS. Technical Consultation on Male Circumcision and HIV Prevention: Research Implications for Policy and Programming. New Data Male Circumcision HIV Prev Policy Programme Implic. 2007; Available: http://www.who.int/hiv/mediacentre/MCrecommendations_en.pdf

[5] WHO-UNAIDS. Joint Strategic Action Framework to Accelerate the Scale-Up of Voluntary Medical Male Circumcision for HIV Prevention in Eastern and Southern Africa 2012–2016 [Internet]. Geneva; 2011. Available: http://www.who.int/hiv/pub/strategic_action2012_2016/en/

[6] WHO-UNAIDS. UNAIDS World AIDS day report [Internet]. Geneva; 2015. Available: http://www.unaids.org/sites/default/files/media_asset/WAD2015_report_en_part01.pdf

[7] Auvert B, Taljaard D, Rech D, Lissouba P, Singh B, Bouscaillou J, et al Association of the ANRS-12126 male circumcision project with HIV levels among men in a South African township: evaluation of effectiveness using cross-sectional surveys. PLoS Med. 2013;10: e1001509 10.1371/journal.pmed.1001509 24019763 PMC3760784

[8] National Department of Health P. The 2010 National antenatal sentinel HIV and syphilis prevalence survey in South Africa. 2011 pp. 50–53.

[9] Vaughan Liwen. Statistical Methods for the Information Professional Second edition New jersy, USA: Information Today, Inc; 2003.

[10] Clopper C, Pearson ES. The use of confidence or fiducial limits illustrated in the case of the binomial. Biometrika. 1934;26: 404–413. 10.1093/biomet/26.4.404

[11] R Development Core Team. R: a language and environment for statistical computing R Foundation for Statistical Computing Vienna, Austria; 2005.

[12] Lissouba P, Taljaard D, Rech D, Doyle S, Shabangu D, Nhlapo C, et al A model for the roll-out of comprehensive adult male circumcision services in African low-income settings of high HIV incidence: the ANRS 12126 Bophelo Pele Project. PLoS Med. 2010;7: e1000309 10.1371/journal.pmed.1000309 20652013 PMC2907271

[13] Mahler HR, Kileo B, Curran K, Plotkin M, Adamu T, Hellar A, et al Voluntary Medical Male Circumcision: Matching Demand and Supply with Quality and Efficiency in a High-Volume Campaign in Iringa Region, Tanzania. Sansom SL, editor. PLoS Med. 2011;8: e1001131 10.1371/journal.pmed.1001131 22140366 PMC3226544

[14] Westercamp N, Bailey RC. Acceptability of Male Circumcision for Prevention of HIV/AIDS in Sub-Saharan Africa: A Review. AIDS Behav. 2007;11: 341–355. 10.1007/s10461-006-9169-4 17053855 PMC1847541

[15] WHO-UNAIDS. Voluntary medical male circumcision for HIV prevention in 14 priority African countries in East and Southern Africa. [Internet]. Geneva; 2015. Report No.: WHO / HIV/2015.21. Available: http://apps.who.int/iris/bitstream/10665/179933/1/WHO_HIV_2015.21_eng.pdf?ua=1&ua=1

[16] Kong X, Ssekasanvu J, Kigozi G, Lutalo T, Nalugoda F, Serwadda D, et al Male circumcision coverage, knowledge, and attitudes after 4-years of program scale-up in Rakai, Uganda. AIDS Behav. 2014;18: 880–884. 10.1007/s10461-014-0740-0 24633740

[17] Ssekubugu R, Leontsini E, Wawer MJ, Serwadda D, Kigozi G, Kennedy CE, et al Contextual Barriers and Motivators to Adult Male Medical Circumcision in Rakai, Uganda. Qual Health Res. 2013;23: 795–804. 10.1177/1049732313482189 23515302

[18] Hatzold K, Mavhu W, Jasi P, Chatora K, Cowan FM, Taruberekera N, et al Barriers and Motivators to Voluntary Medical Male Circumcision Uptake among Different Age Groups of Men in Zimbabwe: Results from a Mixed Methods Study. Tang JW, editor. PLoS ONE. 2014;9: e85051 10.1371/journal.pone.0085051 24802746 PMC4011705

[19] Westercamp N, Bailey RC. Acceptability of Male Circumcision for Prevention of HIV/AIDS in Sub-Saharan Africa: A Review. AIDS Behav. 2006; 10.1007/s10461-006-9169-4 PMC1847541 17053855

[20] Herman-Roloff A, Llewellyn E, Obiero W, Agot K, Ndinya-Achola J, Muraguri N, et al Implementing Voluntary Medical Male Circumcision for HIV Prevention in Nyanza Province, Kenya: Lessons Learned during the First Year. Gray R, editor. PLoS ONE. 2011;6: e18299 10.1371/journal.pone.0018299 21483697 PMC3070734

[21] Jones D, Cook R, Arheart K, Redding CA, Zulu R, Castro J, et al Acceptability, Knowledge, Beliefs, and Partners as Determinants of Zambian Men’s Readiness to Undergo Medical Male Circumcision. AIDS Behav. 2014;18: 278–284. 10.1007/s10461-013-0530-0 23757123 PMC3815686

[22] Thirumurthy H, Masters SH, Rao S, Bronson MA, Lanham M, Omanga E, et al Effect of providing conditional economic compensation on uptake of voluntary medical male circumcision in Kenya: a randomized clinical trial. JAMA. 2014;312: 703–711. 10.1001/jama.2014.9087 25042290 PMC4268484

[23] Lundahl B, Burke BL. The effectiveness and applicability of motivational interviewing: a practice-friendly review of four meta-analyses. J Clin Psychol. 2009;65: 1232–1245. 10.1002/jclp.20638 19739205

[24] Miller WR, Rose GS. Toward a theory of motivational interviewing. Am Psychol. 2009;64: 527–537. 10.1037/a0016830 19739882 PMC2759607

